# Sandflies (Diptera, Psychodidae) from forest areas in Botucatu municipality, central western São Paulo State, Brazil

**DOI:** 10.1186/1678-9199-19-15

**Published:** 2013-06-13

**Authors:** André Antonio Cutolo, Eunice Aparecida Bianchi Galati, Claudio José Von Zuben

**Affiliations:** 1 Division of Health Surveillance, Monte Mor Department of Health, Monte Mor, São Paulo State, Brazil; 2 Department of Epidemiology, School of Public Health, University of São Paulo (USP), São Paulo, São Paulo State, Brazil; 3 Department of Zoology, Rio Claro Biosciences Institute, São Paulo State University (UNESP-Univ Estadual Paulista), Rio Claro, São Paulo State, Brazil

**Keywords:** Cutaneous leishmaniasis, Vector, Sandfly, Brazilian savannah, Semideciduous forest, Cuesta

## Abstract

**Background:**

The study of the distribution and ecology of sandfly species is essential for epidemiological surveillance and estimation of the transmission risk of *Leishmania* spp. infection.

**Findings:**

In the present study, sandflies were captured in native fragmented forest areas in Rubião Júnior district, Botucatu municipality, São Paulo state, Brazil, between September 2001 and January 2005. A minimum of two automatic light traps were installed per night from 6 pm to 8 am, in different months, resulting in approximately 900 collecting hours. During this period, 216 sandfly specimens of sixteen species were captured. *Pintomyia monticola* and *Brumptomyia guimaraesi* were the most abundant with 56 specimens (25.93%) captured per species, followed by *Pintomyia fischeri* 28 (12.96%) and *Psathyromyia pascalei* 18 (8.33%). Other captured species were *Lutzomyia amarali, Sciopemyia sordellii, Psathyromyia aragaoi*, *Nyssomyia whitmani, Migonemyia migonei, Pintomyia bianchigalatiae, Pintomyia misionensis, Brumptomyia carvalheiroi*, *Brumptomyia cardosoi, Brumptomyia cunhai, Brumptomyia nitzulescui, Brumptomyia brumpti* and *Brumptomyia* spp*.* represented by 58 (26.85%) specimens.

**Conclusions:**

Although less frequently found, the presence of *Pintomyia fischeri, Nyssomyia whitmani* and *Migonemyia migonei*, known vectors of *Leishmania braziliensis,* indicates risk of American cutaneous leishmaniasis occurrence. Moreover, the absence of *Lutzomyia longipalpis*-the main vector of *Leishmania infantum chagasi,* which is the agent of American visceral leishmaniasis-suggests that there is no risk of introduction and establishment of this disease in the studied area.

## Findings

Sandflies are vectors of *Leishmania* protozoa, the causative agents of leishmaniases in mammalian hosts. The disease is transmitted through the bite of infected insects when feeding on blood of wild or domestic mammals and even humans in zoonotic sylvatic or peridomestic cycles [1].

Currently, there are more than 900 described species of sandflies, of which approximately 500 are found in the Americas, and more specifically 69 in São Paulo state [2]. Among these species, there are important vectors of *Leishmania* (*Viannia*) *braziliensis-*one of the agents of American cutaneous leishmaniasis (ACL)-such as *Nyssomyia neivai*, *Nyssomyia intermedia*, *Nyssomyia whitmani*, *Migonemyia migonei*, *Pintomyia fischeri* and *Pintomyia pessoai* and the main vector of *Leishmania infantum chagasi* that causes American visceral leishmaniasis (AVL), *Lutzomyia longipalpis*[1].

From 1999 to 2011, a total of 1,927 human cases of AVL, out of which 169 were fatal, were recorded in 75 municipalities of São Paulo state, mainly in the western region, by the São Paulo Health Department [3]. The vector *L. longipalpis* has been found in 108 out of the 645 cities of the state [4]. Between 1998 and September 2010, 7,674 cases of human ACL were recorded in the region in more than 400 municipalities [5].

The understanding of the distribution and ecology of sandflies is essential for leishmaniasis prevention. Botucatu municipality-in São Paulo state-has the same type of vegetation and soil as Itirapina, Ipeúna and Analândia, where the presence of *L. longipalpis* was observed in rural areas in which native vegetation is associated with rock outcrops of basaltic arenite, common in basaltic *cuestas*[6,7].

Additionally, there is evidence that AVL have previously expanded its range along routes of human activity towards western and central western São Paulo state, affecting mainly, though not exclusively, cities close to Marechal Rondon highway (officially designated SP-300) [8]. Consequently, autochthonous cases of human AVL were recorded in the following cities: Araçatuba (1999), Penápolis (2001), Bauru (2003), Agudos (2006) and Lençóis Paulista (2007); which also motivated the investigation of the risks of AVL occurrence in the municipality of Botucatu, because it is on the route of the disease expansion [3].

Two forest fragments situated in the north region of Botucatu-in rural areas of Rubião Júnior district-were considered for sampling. The first fragment (22º 54’ 32” S, 48° 30’33” W, at 845 m above sea level), of approximately 1 hectare, comprises narrow riparian cerrado (Brazilian savannah) with *Stryphnodendron* spp. shrubs, surrounded by pasture. The second fragment (22°55’49” S, 48°32’68” W, at 800 m a.s.l.) is a reserve area of seasonal semideciduous forest, typical of inland Atlantic Forest, of more than 10 hectares, with adult jequitibá trees (*Cairiniana legalis*) and surrounded by pasture. Sampling point coordinates were recorded with a GPS Garmin eTrex™ device.

A minimum of two automatic light traps were placed 30–50 cm above the ground and approximately 50 m apart, on each of the 28 sampling nights. Traps were active during continuous periods from 6 pm to 8 am, on randomly selected days, between September 2001 and January 2005, resulting in approximately 900 hours of sampling. The cerrado area was sampled from September 2001 to April 2003, in December 2004 and January 2005, while the Atlantic Forest area was sampled between August and November 2004. Insects were killed by freezing, macerated, dyed, set on glass slides and identified according to Galati [9].

A total of 216 individuals belonging to 16 species were captured in the study period (Table 1).

**Table 1:** Total number of sandflies, by species and gender, captured with automatic light traps in areas of Atlantic Forest and cerrado, between September 2001 and January 2005, in Rubião Júnior, Botucatu municipality, São Paulo state, Brazil

	**Cerrado**		**Atlantic Forest**		**TOTAL**	
**Species**	**Male**	**Female**	**Male**	**Female**	**Male + Female**	**%**
*Pintomyia monticola*	3	10	5	38	56	25.93
*Pintomyia fischeri*	1	–	12	15	28	12.96
*Psathyromyia pascalei*	–	–	5	13	18	8.33
*Sciopemyia sordellii*	–	1	1	–	2	0.93
*Lutzomyia amarali*	1	1	–	–	2	0.93
*Psathyromyia aragaoi*	–	–	1	–	1	0.46
*Nyssomyia whitmani*	–	–	1	–	1	0.46
*Migonemyia migonei*	–	–	–	1	1	0.46
*Pintomyia misionensis*	–	1	–	–	1	0.46
*Pintomyia bianchigalatiae*	–	1	–	–	1	0.46
*Brumptomyia guimaraesi*	16	16	21	3	56	25.93
*Brumptomyia carvalheiroi*	–	–	9	3	12	5.56
*Brumptomyia cardosoi*	–	–	6	–	6	2.78
*Brumptomyia cunhai*	–	–	3	1	4	1.85
*Brumptomyia nitzulescui*	–	–	2	1	3	1.39
*Brumptomyia brumpti*	–	–	3	–	3	1.39
*Brumptomyia* spp.^*^	–	–	–	21	21	9.72
Total per gender	21	30	69	96	216	100.00
Total	51		165		216	100.00

*damaged individuals, not allowing species identification.

*Pintomyia monticola* was more frequent among the samples; a total of 56 (25.93%) individuals were captured at the sampling points. This species is considered anthropophilic and is suspected of being a vector of *Leishmania enriettii,* the causative agent of cutaneous leishmaniasis in guinea pigs [10].

Out of the total number of captured insects, 105 (48.61%) individuals belonged to the *Brumptomyia* genus, with six species in the area and *Brumptomyia guimaraesi* as the prevalent species in the period, with 56 (25.93%) individuals collected. This genus of sandfly does not have epidemiological importance in the transmission of *Leishmania* spp. and it is known for feeding on blood of armadillos (Dasipodidae: Edentata), mammals occasionally spotted in the sampling points [11]. The high diversity of *Brumptomyia* species can be explained by the presence of an ecotone in the area, the transition area between cerrado and Atlantic Forest vegetation, increasing local biodiversity. *Brumptomyia carvalheiroi* was recorded for the first time outside its typical location [2].

There are no published data in the literature on sandfly entomofauna in Botucatu [2]. However, in the period from 1995 to 2002 four sandfly collections were conducted by the Superintendence for Endemic Disease Control (SUCEN) of São Paulo state in this municipality, when the species *M. migonei, P. fischeri, P. pessoai* and *N. whitmani* were observed, all implicated as vectors in ACL epidemiology.

It is vital to highlight the recording of three vector species of *Leishmania* (*Viannia*) *braziliensis*: *P. fischeri, N. whitmani* and *M. migonei*. Although only one specimen was captured of the two last species, *P. fischeri* was the third most abundant species, with 28 (12.9%) specimens. These three anthropophilic sandfly species are sylvatic [12]. As areas with preserved vegetation in São Paulo state are getting scarce, they are losing importance in the ACL epidemiology, whereas *Nyssomyia intermedia s. lat*.-more adapted to human altered landscapes-has become the main vector of this parasite [12,13].

Only four sandfly species were recorded in the two forest fragments evaluated: *Pintomyia monticola, P. fischeri, Sciopemyia sordellii* and *Brumptomyia guimaraesi*. In the cerrado fragment, three species were exclusive: *Lutzomyia amarali, Pintomyia misionensis* and *Pintomyia bianchigalatiae*. Species found only in the Atlantic Forest area were: *Psathyromyia pascalei, Psathyromyia aragaoi, N. whitmani, M. migonei, Brumptomyia cardosoi, B. nitzulescui, B. avellari, B. carvalheiroi* and *B. cunhai*.

During the evaluated period, *L. longipalpis* was not recorded. The absence of this species indicates no risk of introduction and settlement of AVL in the studied rural area.

Botucatu municipality comprises three distinct areas of diverse altitudes, varying from 400 to 500 meters at the lower part (Peripheral Depression) and from 800 to 900 at the upper part of its territory (Western Plateau), with the basaltic cuestas as transition between the two formations.

Even though the present study included dry and rainy seasons, the results are considered preliminary. For a better evaluation of the faunistic composition of sandflies, precise determination of species dominance and densities, and occasional natural infections by *Leishmania*, a more comprehensive study is required, with more systematic and periodic samplings, including collections at the Peripheral Depression and basaltic cuestas, where different vegetation from the study area can be found, including rock outcrops associated with semidecidual Atlantic Forest, a potential natural habitat for *L. longipalpis*.

It is also important to emphasize the presence of *P. fischeri, N. whitmani* and *M. migonei* in the study area, indicating risk of *L.* (*Viannia*) *braziliensis* transmission to humans in periods in which the enzootic cycles of protozoa are occurring.

## Competing interests

The authors declare that there are no competing interests.

## Authors’ contributions

AAC participated in the design of the study, carried out the insect collection, laboratory preparation, taxonomic identification of sandflies and article writing. EABG participated in the design of the study, took part in the taxonomic identification of sandflies and article writing. CJVZ participated in the discussion of the results obtained and article writing. All authors read and approved the final manuscript.
